# Glycemic control and clinical outcomes in diabetic patients with heart failure and reduced ejection fraction: insight from ventricular remodeling using cardiac MRI

**DOI:** 10.1186/s12933-024-02243-w

**Published:** 2024-04-29

**Authors:** Ke Shi, Ge Zhang, Hang Fu, Xue-Ming Li, Yue Gao, Rui Shi, Hua-Yan Xu, Yuan Li, Ying-Kun Guo, Zhi-Gang Yang

**Affiliations:** 1 Department of Radiology, Functional and Molecular Imaging Key Laboratory of Sichuan Province, West China Hospital, Sichuan University, Chengdu, Sichuan China; 2 Laboratory of Cardiovascular Diseases, Regenerative Medicine Research Center, West China Hospital, Sichuan University, Chengdu, Sichuan China; 3 Department of Radiology, Key Laboratory of Birth Defects and Related Diseases of Women and Children of Ministry of Education, West China Second University Hospital, Sichuan University, Chengdu, Sichuan China

**Keywords:** Glycosylated hemoglobin, Type 2 diabetes mellitus, Heart failure with reduced ejection fraction, Myocardial contractile dysfunction, Outcomes

## Abstract

**Background:**

Glycemic control, as measured by glycosylated hemoglobin (HbA1c), is an important biomarker to evaluate diabetes severity and is believed to be associated with heart failure development. Type 2 diabetes mellitus (T2DM) and heart failure with reduced ejection fraction (HFrEF) commonly coexist, and the combination of these two diseases indicates a considerably poorer outcome than either disease alone. Therefore, glycemic control should be carefully managed. The present study aimed to explore the association between glycemic control and clinical outcomes, and to determine the optimal glycemic target in this specific population.

**Methods:**

A total of 262 patients who underwent cardiac MRI were included and were split by HbA1c levels [HbA1c < 6.5% (intensive control), HbA1c 6.5-7.5% (modest control), and HbA1c > 7.5% (poor control)]. The biventricular volume and function, as well as left ventricular (LV) systolic strains in patients in different HbA1c categories, were measured and compared. The primary and secondary outcomes were recorded. The association of different HbA1c levels with adverse outcomes was assessed.

**Results:**

Despite similar biventricular ejection fractions, both patients with intensive and poor glycemic control exhibited prominent deterioration of LV systolic strain in the longitudinal component (*P* = 0.004). After a median follow-up of 35.0 months, 55 patients (21.0%) experienced at least one confirmed endpoint event. Cox multivariable analysis indicated that both patients in the lowest and highest HbA1c categories exhibited a more than 2-fold increase in the risk for primary outcomes [HbA1c < 6.5%: hazard ratio (HR) = 2.42, 95% confidence interval (CI) = 1.07–5.45; *P* = 0.033; HbA1c > 7.5%: HR = 2.24, 95% CI = 1.01–4.99; *P* = 0.038] and secondary outcomes (HbA1c < 6.5%: HR = 2.84, 95% CI = 1.16–6.96; *P* = 0.022; HbA1c > 7.5%: HR = 2.65, 95% CI = 1.08–6.50; *P* = 0.038) compared with those in the middle HbA1c category.

**Conclusions:**

We showed a U-shaped association of glycemic control with clinical outcomes in patients with T2DM and HFrEF, with the lowest risk of adverse outcomes among patients with modest glycemic control. HbA1c between 6.5% and 7.5% may be served as the optimal hypoglycemic target in this specific population.

## Introduction

Type 2 diabetes mellitus (T2DM) is one of the most common comorbidities of heart failure with reduced ejection fraction (HFrEF) due to the interaction between the two diseases. It has been reported that T2DM impacts one-third of all patients in the general heart failure (HF) population, with its prevalence gradually increasing in the last decade [1, 2]. Previous studies have established diabetic status as an independent predictor of worse outcomes in HFrEF, and glycemic control is considered to be associated with HF development [1, 3, 4]. Data from clinical trials and multicenter studies have suggested that improved glycemic control, as measured by glycosylated hemoglobin (HbA1c), has the potential to reduce the risk of major microvascular or macrovascular complications in T2DM patients and thus improve their long-term survival rate [5–7]. However, for patients with coexisting T2DM and HFrEF, glycemic control should be carefully managed since the combination of these two diseases indicates a considerably poorer outcome than either disease alone. To date, no agreement has been reached regarding the optimal range of glycemic control in T2DM patients with HFrEF [8–11]. Furthermore, the influence of glycemic control on myocardial systolic dysfunction in this specific population remains unknown. In view of the question of whether glycemic control matters in T2DM patients with HFrEF, the present study was therefore conducted to explore what the risk did different HbA1c categories convey. We evaluated biventricular volume and function, as well as left ventricular (LV) myocardial contractility using cardiac magnetic resonance imaging (MRI), and sought to determine the association between specific HbA1c levels and outcomes, and what range of HbA1c defines optimum control in T2DM patients with HFrEF.

## Methods

### Study cohort

Patients with HFrEF referred to our hospital from January 2015 to August 2022 were screened for inclusion in this study. The diagnosis of HFrEF was established according to the guidelines from the European Society of Cardiology (2021). All patients had to meet the following criteria: (1) presence of symptoms and/or signs of HF for more than 3 months; (2) an elevated amino-terminal pro-B-type natriuretic peptide (NT-proBNP) level; and (3) a reduced LV ejection fraction (LVEF ≤ 40%). The diagnosis of DM was made based on the current guidelines from the European Society of Cardiology (2019) [12]. Patients were excluded if they met at least one of the following criteria: (1) age younger than 18 years; (2) acute coronary syndrome; (3) severe arrhythmia; or (4) incomplete clinical or MRI information. Finally, a total of 262 patients satisfied the abovementioned criteria and were enrolled as the study cohort. Demographics, clinical characteristics, laboratory measurements and medical treatments at baseline were retrieved from a review of hospital records. Patients were subdivided into 3 groups according to their HbA1c levels: HbA1c < 6.5% (intensive control), HbA1c 6.5-7.5% (modest control), and HbA1c > 7.5% (poor control). This study was approved by the Biomedical Research Ethics Committees of our hospital and complied with the Declaration of Helsinki. All medical data were protected with full confidentiality and used only for the purpose of the present study.

Patient follow-up was continued until September 2023 by reviewing electronic medical records or telephone interviews. The duration of follow-up was calculated as the time from cardiac MRI to either the occurrence of any endpoint or the last follow-up date. The primary endpoint was the composite of HF hospitalization, cardiovascular mortality and heart transplantation, whichever occurred first. The secondary endpoint was HF hospitalization. HF hospitalization was defined as an unplanned hospitalization or an urgent hospital visit for worsening HF.

### Imaging acquisition and postprocessing

Cardiac MRI was performed on a 3-Tesla scanner (MAGNETOM Skyra/Tim Trio; Siemens Healthcare, Erlangen, Germany) for each patient to acquire cardiac structure and function parameters. Cine images were obtained during breath-holding at end-expiration using a balanced steady-state free precession (SSFP) sequence [repetition time (TR) = 2.81 ms; time to echo (TE) = 1.22 ms; slice thickness = 8.0 mm; flip angle (FA) = 40°/50°; acquisition matrix = 166 × 208 pixels; and field of view (FOV) = 340 × 284 mm^2^]. Approximately 10–15 short-axis images from base to apex were obtained, as well as 4-, 2- and 3-chamber long-axis images.

All images were analyzed using commercially available CVI^42^ software (Circle Cardiovascular Imaging, Inc., Calgary, Alberta, Canada) or at Siemens Argus workstation. For volumetric analyses, endocardial and epicardial borders were traced semiautomatically at the end-diastolic and end-systolic phases on the short-axis stacks and manually corrected if needed. biventricular function parameters, including EF, end-diastolic volume (EDV), end-systolic volume (ESV), and stroke volume (SV), were automatically calculated. LV papillary muscles were included in the LV mass (LVM) but not in the LV volume. Biventricular volumetric measurements and LVM were indexed for body surface area. For LV contractility analyses, a stack of short-axis cine images combined with 4-, 2- and 3-chamber long-axis images were loaded into the feature-tracking module. We delineated LV endocardial and epicardial borders at the end-diastolic phase (reference phase) of all cine images. The software automatically traced the contours throughout the cardiac cycle. Global myocardial strain was calculated as the total deformation of the myocardium from its initial length at the end-diastolic phase to its final length at the end-systolic phase and is expressed as a percentage. Positive and negative signs of myocardial strain [peak strain (PS)] indicate shortening and thickening of the myocardium, respectively.

### Statistical analysis

Statistical analyses were performed using SPSS (IBM SPSS Inc., Armonk, New York, USA) and Prism (GraphPad Software Inc., San Diego, California, USA). The normality of the data was determined using the Shapiro–Wilk test. Data are expressed as the means with standard deviations or medians with interquartile ranges (IQRs) for continuous variables and frequencies for categorical variables. Comparative analyses were repeated in subgroups after stratification of the study cohort into 3 categories (HbA1c < 6.5%, HbA1c 6.5-7.5%, and HbA1c > 7.5%) using one-way analysis of variance, followed by Bonferroni post hoc correction or its nonparametric equivalent (Kruskal–Wallis test), chi–square test or Fisher’s exact test, as appropriate. The primary and secondary endpoints were assessed using Kaplan–Meier survival analysis and compared among different categories of HbA1c with the log-rank test. Associations between different HbA1c categories and adverse outcomes were determined using a multivariable Cox proportional hazards model. Significant variables (P value < 0.10) in the univariable model were used to construct the multivariable model predicting either primary or secondary endpoint. HbA1c category was treated as a dummy variable with the middle HbA1c category of 6.5-7.5% as the reference when performing Cox analysis. Differences with a two-tailed P value < 0.05 were considered indicative of statistical significance.

## Results

### Clinical characteristics

The clinical characteristics of the study cohort are summarized in Table 1. Demographic characteristics, HF duration, New York Heart Association (NYHA) functional class, ischemic etiology and medical history were comparable among the three groups. As expected, the proportion of people with a diabetes duration of more than 5 years was highest in the highest HbA1c category (HbA1c > 7.5%), intermediate in the middle HbA1c (6.5-7.5%) category, and lowest in the lowest HbA1c category (HbA1c < 6.5%) (*P* < 0.001). Furthermore, patients in the middle HbA1c category exhibited the lowest level of NT-proBNP compared to patients with HbA1c < 6.5% and > 7.5% (HbA1c < 6.5%: [2449 (1312, 4261) pg/mL] vs. HbA1c of 6.5-7.5%: [2039 (957, 3968) pg/mL] vs. HbA1c > 7.5%: [2833 (1239, 8587) pg/mL]; *P* = 0.02). Patients in the highest HbA1c category had the highest level of fasting blood glucose compared with patients with HbA1c < 6.5% and 6.5%-7.5% (*P* < 0.001).

**Table 1:** Clinical characteristics of the study population according to glycemic control

Variables	HbA1c category		
	< 6.5% (*n* = 73)	6.5-7.5% (*n* = 90)	> 7.5% (*n* = 99)
Age, yrs	55.1 ± 12.2	56.0 ± 11.6	57.9 ± 10.9
Male, n (%)	49 (67.1)	66 (73.3)	70 (70.7)
BMI, kg/m^2^	25.4 ± 4.0	24.9 ± 3.8	23.8 ± 3.4
SBP, mmHg	118.7 ± 19.7	119.1 ± 19.9	123.6 ± 22.2
DBP, mmHg	80.2 ± 14.9	77.7 ± 14.9	79.7 ± 15.0
HR, beats/min	87.8 ± 18.5	85.4 ± 19.6	86.5 ± 14.0
Smoking, n (%)	36 (49.3)	45 (50.0)	49 (49.5)
Drinking, n (%)	30 (41.1)	31 (34.4)	32 (32.3)
HF duration, n (%)			
≤ 1 yr	41 (56.2)	49 (54.5)	51 (51.5)
> 1 and ≤ 5 yrs	19 (26.0)	31 (34.4)	28 (28.3)
> 5 yrs	13 (17.8)	10 (11.1)	20 (20.2)
NYHA functional class III–IV, n (%)	61 (83.6)	81 (90.0)	88 (88.9)
Ischemic etiology, n (%)	23 (31.5)	30 (33.3)	27 (27.3)
DM duration, n (%)			
≤ 1 yr	54 (74.0)	45 (50.0) ^§^	35 (35.3) ^§λ^
> 1 and ≤ 5 yrs	9 (12.3)	16 (17.8)	18 (18.2)
> 5 yrs	10 (13.7)	29 (32.2) ^§^	46 (46.5) ^§λ^
Medical history^a^, n (%)			
HT	30 (41.1)	45 (50.0)	52 (52.5)
AF	13 (17.8)	24 (26.7)	15 (15.2)
Dyslipidemia	26 (35.6)	30 (33.3)	45 (45.5)
LBBB	7 (9.6)	5 (5.6)	9 (9.1)
Laboratory measurements			
NT‑proBNP, pg/mL	2449 (1312, 4261)	2039 (957, 3968) ^&^	2833 (1239, 8587) ^#^
eGFR, mL/min/1.73m^2^	72.3 ± 26.6	77.3 ± 21.9	70.1 ± 24.7
FBG, mmol/L	6.5 (5.2, 8.3)	7.0 (5.9, 8.7) ^&^	9.5 (7.4, 12.8) ^&#^
HbA1c, %	6.0 (5.7, 6.3)	6.9 (6.7, 7.2) ^&^	8.5 (8.0, 9.5) ^&#^
Hemoglobin, g/L	135.6 ± 24.7	141.4 ± 25.5	135.3 ± 23.4
Cardiovascular medications, n (%)			
Beta‑blocker	53 (72.6)	72 (80.0)	79 (79.8)
ACEI/ARB	61 (83.6)	69 (76.7)	69 (69.7)
ARNI	40 (54.8)	46 (51.1)	49 (49.5)
SGLT-2i	26 (35.6)	30 (33.3)	32 (32.3)
Loop diuretics	57 (78.1)	69 (76.7)	73 (73.7)
MRA	55 (75.3)	71 (78.9)	71 (71.7)
Anti-thrombotic agents^b^	34 (46.6)	46 (51.1)	64 (64.6) ^§λ^
Statins	25 (34.2)	45 (50.0) ^§^	57 (57.6) ^§^
Hypoglycemic medications, n (%)			
Insulin	13 (17.8)	25 (27.8)	46 (46.5) ^§λ^
Metformin	20 (27.4)	27 (30.0)	47 (47.5) ^§λ^
Sulfonylureas	9 (12.3)	15 (16.7)	15 (15.2)
α-Glucosidase inhibitors	21 (28.8)	26 (28.9)	35 (35.4)

Data are presented as mean ± SD, media (Q1, Q3) or number (percentage)

Kruskal-Wallis test: ^&^ P-value < 0.05 versus category of HbA1c < 6.5%. ^#^ P-value < 0.05 versus category of HbA1c 6.5-7.5%. Chi-square test (Fisher’s exact test): ^§^ P-value < 0.05 versus category of HbA1c < 6.5%. ^λ^ P-value < 0.05 versus category of HbA1c 6.5-7.5%

^a^. The diagnosis was made based on the clinical evaluation at our institute

^b^. Patients with self-reported history of AF, surgical valve replacement or thrombosis history were prescribed anti-thrombotic agents

Abbreviations: HbA1c, glycated hemoglobin; BMI, body mass index; SBP, systolic blood pressure; DBP, diastolic blood pressure; HR, heart rate; HF, heart failure; NYHA, New York Heart Association; DM, diabetic mellitus; HT, hypertension; AF, atrial fibrillation; LBBB, complete left bundle branch block; NT-proBNP, amino-terminal pro-B-type natriuretic peptide; eGFR, estimated glomerular filtration rate; FBG, fasting blood glucose; ACEI, angiotensin converting enzyme inhibitor; ARB, angiotensin receptor blocker; ARNI, angiotensin receptor-neprilysin inhibitor; SGLT-2i, sodium-glucose cotransporter-2 inhibitors; MRA, mineralocorticoid receptor antagonist; α-GI, α-Glucosidase inhibitors

The use of cardiovascular medications was similar among the patients in the three categories of HbA1c, except antithrombotic agents (*P* = 0.04) and statins (*P* = 0.01), which were more likely to be prescribed to patients in the highest HbA1c category. With respect to hypoglycemic medications, there were more patients in the highest HbA1c category treated with insulin (*P* < 0.001) and metformin (*P* = 0.009) than those in the lower two categories of HbA1c < 6.5% and 6.5%-7.5%.

### Cardiac MRI findings

**Fig. 1:**
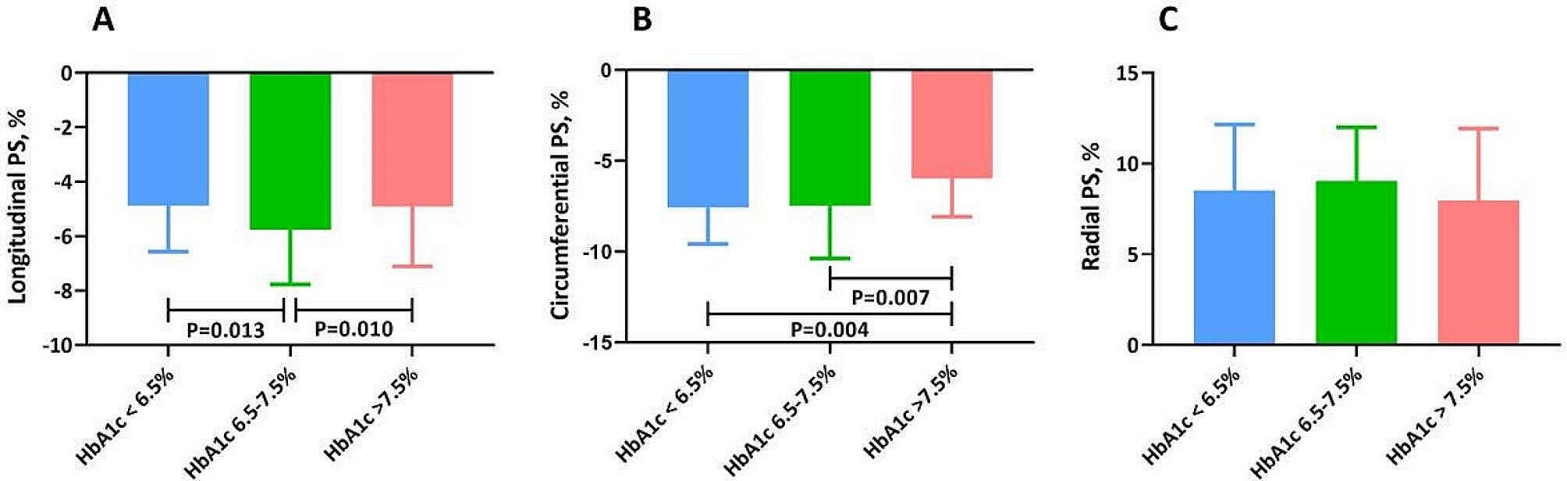
Differences of magnitude of global left ventricular longitudinal (**A**), circumferential (**B**) and radial (**C**) PS across the groups. Abbreviations: PS, peak strain

As shown in Table 2, similar LV sizes (LV end-diastolic volume (LVEDV) and LV end-systolic volume (LVESV)) and LVM were observed across the three groups by HbA1c category. There was a trend toward a lower LV stroke volume (LVSV) (*P* = 0.08) and LVSV index (LVSVi) (*P* = 0.09) in the highest category of HbA1c than in the other two categories of HbA1c. The percentage of mitral regurgitation was comparable among the three groups. Although there was no significant difference in LVEF across the different HbA1c categories, deterioration of the magnitude of longitudinal PS was more prominent in both the lowest and highest HbA1c categories compared to that in the middle HbA1c category (HbA1c < 6.5%: -4.9 ± 1.7% vs. HbA1c of 6.5-7.5%: -5.8 ± 2.0% vs. HbA1c > 7.5%: -4.9 ± 2.2%; *P* = 0.004). Moreover, more severe impairment in the magnitude of circumferential PS was demonstrated in patients in the highest HbA1c category than in those in the lowest or middle HbA1c category [HbA1c < 6.5%: -7.6% (-5.3%, -9.6%) vs. HbA1c of 6.5-7.5%: -7.5% (-5.9%, -10.4%) vs. HbA1c > 7.5%: -6.0% (-4.9%, -8.1%); *P* = 0.005]. Nevertheless, no significant difference was found in radial PS [HbA1c < 6.5%: 8.5% (6.5%, 12.2%) vs. HbA1c of 6.5-7.5%: 9.0% (5.8%, 12.0%) vs. HbA1c > 7.5%: 8.0% (5.1%, 11.9%); *P* = 0.313] (Fig. 1). We also recorded the right ventricular volume and function of the entire cohort and found no difference across the HbA1c category.

**Table 2:** Cardiac MRI findings by HbA1c category

Variables	HbA1c category		
	< 6.5% (*n* = 73)	6.5-7.5% (*n* = 90)	> 7.5% (*n* = 99)
LVEDV, mL	250.1 (190.1, 310.1)	252.8 (190.7, 306.2)	244.7 (194.2, 314.6)
LVEDV index, mL/m^2^	144.8 (115.2, 174.6)	146.1 (116.8, 176.1)	151.6 (120.8, 188.4)
LVESV, mL	174.5 (135.7, 241.4)	192.5 (129.7, 238.1)	193.8 (128.1, 259.7)
LVESV index, mL/m^2^	95.6 (83.1, 134.5)	112.7 (80.0, 136.0)	116.2 (80.3, 147.7)
LVSV, mL	62.2 (47.0, 75.1)	59.5 (43.6, 75.4)	54.4 (41.6, 72.1)
LVSV index, mL/m^2^	35.9 (28.8, 44.5)	35.3 (26.9, 45.3)	31.9 (23.9, 40.1)
LVEF, %	27.8 (19.7, 31.1)	25.3 (17.8, 32.9)	23.1 (16.1, 31.1)
LVM, g	132.8 (117.6, 151.8)	132.5 (116.9, 161.9)	137.9 (118.7, 158.0)
LVM index, g/m^2^	76.9 (65.3, 91.2)	78.7 (67.2, 94.0)	84.2 (69.8, 93.9)
MR, n (%)	39 (53.4)	42 (46.7)	51 (51.5)
LV longitudinal PS, %	-4.9 ± 1.7	-5.8 ± 2.0^*^	-4.9 ± 2.2^†^
LV circumferential PS, %	-7.6 (-5.3, -9.6)	-7.5 (-5.9, -10.4)	-6.0 (-4.9, -8.1) ^&#^
LV radial PS, %	8.5 (6.5, 12.2)	9.0 (5.8, 12.0)	8.0 (5.1, 11.9)
RVEDV, mL	126.2 (91.2, 170.8)	130.8 (102.4, 172.3)	129.8 (94.3, 172.0)
RVEDV index, mL/m^2^	74.3 (54.2, 94.6)	75.4 (58.8, 107.3)	76.5 (53.3, 100.3)
RVESV, mL	64.9 (44.9, 106.5)	81.7 (60.7, 123.7)	78.5 (49.5, 119.9)
RVESV index, mL/m^2^	43.6 (27.7, 56.8)	47.4 (33.5, 70.7)	46.6 (31.2, 69.5)
RVSV, mL	50.8 ± 19.6	48.3 ± 17.6	44.1 ± 20.4
RVSV index, mL/m^2^	29.5 ± 10.6	28.2 ± 9.9	25.8 ± 12.0
RVEF, %	35.5 ± 16.7	36.2 ± 12.9	40.6 ± 14.1

Data are presented as mean ± SD, media (Q1, Q3) or number (percentage)

One-way analysis of variance test: ^*^ P-value < 0.017 versus category of HbA1c < 6.5%. ^†^ P-value < 0.017 versus category of HbA1c 6.5-7.5%. Kruskal-Wallis test: ^&^ P-value < 0.05 versus category of HbA1c < 6.5%. ^#^ P-value < 0.05 versus category of HbA1c 6.5-7.5%

Abbreviations: HbA1c, glycated hemoglobin; LVEDV, left ventricular end-diastolic volume; LVESV, left ventricular end-systolic volume; LVSV, left ventricular stroke volume; LVEF, left ventricular ejection fraction; LVM, left ventricular mass; MR, mitral regurgitation; LV, left ventricular; PS, peak strain; RVEDV, right ventricular end-diastolic volume; RVESV, right ventricular end-systolic volume; RVSV, right ventricular stroke volume; RVEF, right ventricular ejection fraction

### Association between glycemic control and outcomes

**Fig. 2:**
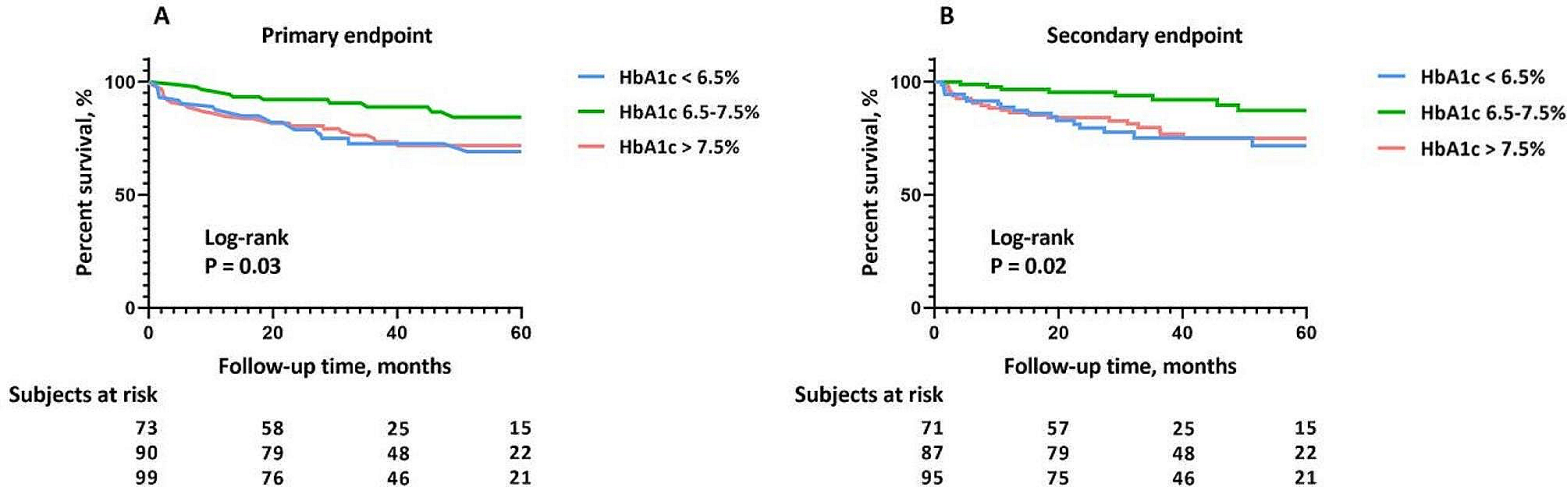
Survival curves of the study cohort for the primary (**A**) and secondary endpoint (**B**) according to glycemic control

During a median follow-up of 35.0 months (Q1-Q3, 22.3–55.5 months), a total of 55 patients (21.0%) experienced at least one confirmed endpoint event, with 46 HF hospitalizations, 5 cardiovascular deaths and 4 heart transplantations. Both primary (26.0% vs. 12.2% vs. 25.3%; *P* = 0.041) and secondary endpoints (23.3% vs. 8.9% vs. 21.2%; *P* = 0.027) occurred less frequently among patients in the middle HbA1c category. In Kaplan–Meier survival analysis, patients in the middle HbA1c category were less likely to experience the primary and secondary outcomes than those in the lowest and highest HbA1c categories during follow-up (log-rank *P* = 0.032 and 0.019, respectively) (Fig. 2).

Tables 3 and 4 showed the univariate Cox regression analysis for the primary and secondary endpoints, respectively. NT-proBNP, insulin and metformin use, HbA1c < 6.5% or > 7.5%, and LV longitudinal PS were associated with adverse outcomes. In the Cox multivariable analysis, HbA1c < 6.5% [hazard ratio (HR) = 2.42; 95% confidence interval (CI), 1.07–5.45; *P* = 0.033] and HbA1c > 7.5% (HR = 2.24; 95% CI, 1.01–4.99; *P* = 0.038) both associated with primary composite endpoint, adjusted to the effects of the other variables in the model. Likewise, when considering the secondary endpoint, HbA1c < 6.5% (HR = 2.84; 95% CI, 1.16–6.96; *P* = 0.022) and HbA1c > 7.5% (HR = 2.65; 95% CI, 1.08–6.50; *P* = 0.038) remained independently associated with the occurrence of HF rehospitalization.

**Table 3:** Cox proportional hazards regression analysis to identify associated variables of primary outcomes

	Univariable analysis		Multivariable analysis	
	HR (95% CI)	P-value	HR (95% CI)	P-value
DM duration > 5 yrs	1.52 (1.09, 2.59)	0.094		
NT‑proBNP^§^	2.69 (1.59, 4.54)	< 0.001	1.78 (1.04, 3.02)	0.034
Insulin use	2.07 (1.23, 3.48)	0.006	1.96 (1.14, 3.40)	0.016
Metformin use	0.48 (0.25, 0.94)	0.032		
HbA1c of 6.5-7.5%	1.00 (reference)		1.00 (reference)	
HbA1c < 6.5%	3.06 (1.38, 6.76)	0.006	2.42 (1.07, 5.45)	0.033
HbA1c > 7.5%	3.22 (1.52, 6.79)	0.002	2.24 (1.01, 4.99)	0.038
LV longitudinal PS^#^	1.28 (1.10, 1.49)	0.002	1.19 (1.01, 1.39)	0.033

^§^. NT-proBNP is log-transformed before being included in the analysis

^#^. LV longitudinal PS is negative value in this analysis

Abbreviations: HR, hazards ratio; CI, confidence interval; DM, diabetic mellitus; NT-proBNP, amino-terminal pro-B-type natriuretic peptide; HbA1c, glycated hemoglobin; LV, left ventricular; PS, peak strain

**Table 4:** Cox proportional hazards regression analysis to identify associated variables of secondary outcomes

	Univariable analysis		Multivariable analysis	
	HR (95% CI)	P-value	HR (95% CI)	P-value
DM duration > 5 yrs	1.41 (1.07, 2.53)	0.091		
NT‑proBNP^§^	2.75 (1.52, 4.98)	0.001	1.73 (1.09, 3.15)	0.041
Insulin use	2.08 (1.19, 3.64)	0.011	1.99 (1.10, 3.62)	0.023
Metformin use	0.40 (0.19, 0.85)	0.017	0.45 (0.21, 0.99)	0.048
HbA1c of 6.5-7.5%	1.00 (reference)		1.00 (reference)	
HbA1c < 6.5%	3.54 (1.47, 8.54)	0.005	2.84 (1.16, 6.96)	0.022
HbA1c > 7.5%	3.57 (1.55, 8.27)	0.003	2.65 (1.08, 6.50)	0.038
LV longitudinal PS^#^	1.29 (1.09, 1.52)	0.003	1.20 (1.01, 1.42)	0.043

^§^. NT-proBNP is log-transformed before being included in the analysis

^#^. LV longitudinal PS is negative value in this analysis

Abbreviations: HR, hazards ratio; CI, confidence interval; DM, diabetic mellitus; NT-proBNP, amino-terminal pro-B-type natriuretic peptide; HbA1c, glycated hemoglobin; LV, left ventricular; PS, peak strain

## Discussion

The main findings of the present study can be summarized as follows: (1) In T2DM patients with concomitant HFrEF, both intensive and poor glycemic control showed more severe impairment in LV myocardial mechanics despite similar biventricular EFs across HbA1c categories. (2) Patients split by levels of HbA1c exhibited a U-shaped relationship with long-term prognosis with an increased risk of worse cardiovascular outcomes for both lower (HbA1c < 6.5%) and higher (HbA1c > 7.5%) levels of HbA1c when compared with those patients with modest glycemic control (HbA1c of 6.5-7.5%). (3) HbA1c range between 6.5% and 7.5% seemed to be the optimum glycemic target.

### Intensive glycemic control and outcomes

It has long been recognized that hyperglycemia has adverse effects on the vasculature, and diabetes treatment guideline recommendations for intensive glucose lowering for T2DM patients have therefore been implemented for decades [13]. Data from recent studies seemingly showed that intensive glycemic control to achieve normal or near-normal HbA1c levels was potentially beneficial to delay the progression of microvascular or macrovascular complications and improve prognosis [14–17]. However, regarding T2DM patients with HFrEF, we found an unexpected risk with intensive glycemic control, which conveyed a more than 2-fold risk of adverse outcomes compared with modest glycemic control. The relationship between intensive glycemic control and cardiovascular outcomes among T2DM patients with concomitant HFrEF has been studied previously, with discrepant results reported [1, 3, 8–11]. Only some of these studies indicated worse outcomes among patients who adopted intensive glucose-lowering strategies, while others showed a lower risk of adverse events or no benefit. Our data support the findings in the former. Moreover, we observed a more pronounced LV contractile dysfunction by cardiac MRI in patients with intensive glycemic control (HbA1c < 6.5%) than in patients with modest glycemic control (HbA1c of 6.5-7.5%). To our knowledge, hypoglycemia-induced cardiac remodeling could be the possible reason behind our findings. In the present study, the prevalence of hypoglycemic medication use in the lowest HbA1c category was no less than that in the middle HbA1c category. Glucose-lowering strategies may be tolerated by diabetic patients but hazardous for those with established HFrEF. Hypoglycemia could induce a broad range of abnormal activation of the sympatho-adrenal system with a surge in catecholamines, resulting in blood and glucose redistribution, augmented cardiac workload, and myocardial ischemia, thereby prompting contractility impairment [8, 18, 19].

### Poor glycemic control and outcomes

In the present study, uncontrolled glycemia as defined by an HbA1c level > 7.5% was also associated with a more severe decline in LV contractile function in patients with T2DM comorbid with HFrEF, with a higher risk of adverse events in comparison with patients with modest glycemic control (HbA1c of 6.5-7.5%). In a sense, these findings were not surprising, but we confirmed a more deteriorated LV dysfunction in these individuals by myocardial strain analysis. The increased risk of cardiovascular events associated with the highest HbA1c level in this study cohort may include both direct and indirect effects of hyperglycemia [20, 21]. Notably, hypoglycemic treatments such as insulin and metformin were more frequently used in patients with poor glycemic control. However, the current findings revealed an independent association between poor glycemic control and adverse outcomes when adjusting for insulin and metformin, which is one major confounder that affects prognosis [22, 23]. Therefore, hyperglycemia itself could accelerate the detrimental process of LV dysfunction and is a biomarker for more advanced or severe stages of HF, which may contribute to adverse events.

### Clinical implications

Our study emphasized the U-shaped relationship between glycemic control and clinical outcomes in patients with T2DM and HFrEF. The optimal target level of HbA1c should be reconsidered in this population since the severity of coexisting HF is important for guiding hypoglycemic strategies and intervention targets in quality HF guideline care. This study, together with previous data, paves the way for future studies to better understand the reciprocal relationship between diabetic status and HF and test a relatively safe treatment strategy targeting lowering HbA1c in this specific population.

### Study limitations

The present study has several limitations. First, due to the limited study population, we stratified the patients into only three categories according to HbA1c levels. Although the current findings were in keeping with previous data, we believe future research with a greater number of HbA1c categories will be more beneficial to identify the optimal treatment goal. Second, the present study found an independent prognostic indication of poor outcomes in both the lowest and highest HbA1c categories of patients after taking hypoglycemic medications into consideration. However, it would be interesting to clarify if there were any differences in the magnitude of the HbA1c lowering effect by different hypoglycemic therapies, especially in those patients in the lowest HbA1c category (i.e., HbA1c < 6.5%). Finally, we must acknowledge that due to the retrospective nature of this study, selection bias was inevitable.

In conclusion, given the U-shaped association of glycemic control with LV remodeling and clinical outcomes in patients with T2DM and HFrEF, we highlight that glycemic control goal with the HbA1c between 6.5% and 7.5% may help to improve the prognosis in this high-risk population. Clinicians should be aware of the possibility of adverse events, especially in those with strict glycemic control (HbA1c < 6.5%), and further studies are needed to verified this optimal treatment goals.

## Data Availability

No datasets were generated or analysed during the current study.

## Abbreviations

T2DM: type 2 diabetes mellitus
HFrEF: heart failure with reduced ejection fraction
HF: heart failure
HbA1c: glycosylated hemoglobin
LV: left ventricular
MRI: magnetic resonance imaging
NT-proBNP: amino-terminal pro-B-type natriuretic peptide
LVEF: left ventricular ejection fraction
SSFP: steady-state free precession
TR: repetition time
TE: time to echo
FA: flip angle
FOV: field of view
LVEDV: left ventricular end-diastolic volume
LVESV: left ventricular end-systolic volume
LVSV: left ventricular stroke volume
LVM: left ventricular mass
PS: peak strain
IQRs: interquartile ranges
NYHA: New York Heart Association
ARNI: angiotensin receptor-neprilysin inhibitor
SGLT-2i: sodium-glucose cotransporter-2 inhibitor
HR: hazard ratio
CI: confidence interval
